# CaWRKY22b Plays a Positive Role in the Regulation of Pepper Resistance to *Ralstonia solanacearum* in a Manner Associated with Jasmonic Acid Signaling

**DOI:** 10.3390/plants13152081

**Published:** 2024-07-27

**Authors:** Lanping Shi, Yuemin Fan, Yingjie Yang, Shuangshuang Yan, Zhengkun Qiu, Zhiqin Liu, Bihao Cao

**Affiliations:** 1 Key Laboratory of Biology and Genetic Improvement of Horticultural Crops (South China), College of Horticulture, South China Agricultural University, Ministry of Agriculture and Rural Affairs, Guangzhou 510642, China; slpfujian@126.com (L.S.); ssyan@scau.edu.cn (S.Y.); qiuzhengkun@scau.edu.cn (Z.Q.); 2 Key Laboratory of Ministry of Education for Genetics, Breeding and Multiple Utilization of Crops, Fujian Agriculture and Forestry University, Fuzhou 350002, China; hi2fanfan@163.com (Y.F.); hnyingjieyang@163.com (Y.Y.); 3 College of Agriculture, Fujian Agriculture and Forestry University, Fuzhou 350002, China

**Keywords:** pepper, WRKY transcription factor, *Ralstonia solanacearum*, immunity, JA signaling

## Abstract

As important transcription factors, WRKYs play a vital role in the defense response of plants against the invasion of multiple pathogens. Though some WRKY members have been reported to participate in pepper immunity in response to *Ralstonia solanacearum* infection, the functions of the majority of WRKY members are still unknown. Herein, *CaWRKY22b* was cloned from the pepper genome and its function against *R. solanacearum* was analyzed. The transcript abundance of *CaWRKY22b* was significantly increased in response to the infection of *R. solanacearum* and the application of exogenous methyl jasmonate (MeJA). Subcellular localization assay in the leaves of *Nicotiana benthamiana* showed that CaWRKY22b protein was targeted to the nuclei. *Agrobacterium*-mediated transient expression in pepper leaves indicated that *CaWRKY22b* overexpression triggered intensive hypersensitive response-like cell death, H_2_O_2_ accumulation, and the up-regulation of defense- and JA-responsive genes, including *CaHIR1*, *CaPO2*, *CaBPR1*, and *CaDEF1*. Virus-induced gene silencing assay revealed that knock-down of *CaWRKY22b* attenuated pepper’s resistance against *R. solanacearum* and the up-regulation of the tested defense- and jasmonic acid (JA)-responsive genes. We further assessed the role of CaWRKY22b in modulating the expression of JA-responsive *CaDEF1*, and the result demonstrated that CaWRKY22b trans-activated *CaDEF1* expression by directly binding to its upstream promoter. Collectively, our results suggest that CaWRKY22b positively regulated pepper immunity against *R. solanacearum* in a manner associated with JA signaling, probably by modulating the expression of JA-responsive *CaDEF1*.

## 1. Introduction

Living in the natural environment, plants are frequently attacked by a wide range of pathogens with different lifestyles, which usually lead to serious diseases in plants. To survive, plants have evolved a complicated and multilayered inducible immune system to fend off pathogens and protect themselves [1]. It is well established that their well-orchestrated immune system can be broadly divided into two layers: PTI (Pathogen-associated molecular pattern (PAMP)-triggered immunity) and ETI (effector-triggered immunity) [1]. For the first layer of defense, pattern-recognition receptors are employed by plants to recognize PAMPs, which are conserved molecules that are found in many different types of pathogens. Examples of PAMPs include bacterial flagellin, fungal chitin, and viral double-stranded RNA [2]. To defend, pathogens can enhance their virulence by secreting effectors that trigger susceptibility in the host, a process known as effector-triggered susceptibility (ETS), which leads to the host’s susceptibility. To prevent PTI, plants have evolved disease resistance (R) proteins to recognize defined effectors directly or indirectly and trigger ETI. PTI and ETI initiate a series of shared defense responses, including the production of reactive oxygen species (ROS), callose deposition at the infection site, and the activation of signaling pathways that lead to the expression of defense-associated genes.

A crucial stage in triggering plant immunity involves the activation of immune responses, which is the transcriptional regulation of defense-associated genes mediated by transcription factors (TFs). TFs regulate the gene expression by binding to the *cis*-elements in the promoter of a target gene. Among plant TFs, WRKY transcription factors are a large family of regulatory proteins. They are characterized by the presence of one or two WRKY domains, which are approximately 60 amino acids in length [3]. The name “WRKY” comes from the conserved amino acid sequence within the domain, encompassing a conserved motif (WRKYGQK) at the N terminus and a zinc-finger motif at the C terminus [3]. The *WRKY* gene families of *Arabidopsis thaliana* (*Arabidopsis*) and *Capsicum annuum* (pepper) consist of 72 and 71 members, respectively [4,5]. Based on the primary structure, WRKY members have been classified into three groups (I, II, and III) and various subgroups (e.g., IIa, IIb, etc.) [3]. It is widely known that WRKY TFs function as transcriptional regulators by directly binding to the W-box or WL-box contained in the promoters of downstream genes, thus activating or suppressing their expression during a variety of immune responses including PTI, ETI, and systemic acquired resistance (SAR) [6]. However, other types of promoter motifs, including the WK-box and sugar-responsive *cis*-element, are also found to be targeted by WRKY TFs and function in the transcriptional expression of target genes [7,8].

WRKY TFs were reported to play a regulatory role in defense response against biotic and abiotic stresses in plants. Additionally, several lines of evidence indicated that WRKY TFs participate in the jasmonic acid (JA) signaling pathway [9,10]. JA is a key phytohormone involved in the defense response of plants against multiple biotic and abiotic stresses. WRKY TFs were found to mediate JA signaling by regulating the expression of downstream genes involved in JA biosynthesis, perception, and signaling transduction [11,12,13,14,15]. *Arabidopsis* WRKY33 positively regulates the resistance of *Arabidopsis* against *Botrytis cinerea* infection and actives JA signaling by repressing the expression of JAZ1, which encodes a repressor protein that negatively regulates JA signaling [16]. *Oryza sativa* OsWRKY45 was reported to act as a negative regulator of JA-mediated defense response, practically by interacting with the JA receptor CORONATINE INSENSITIVE1 (COI1) [17]. The roles of WRKY TFs in JA signaling were well explored in model plants, including *Arabidopsis* and rice. Nevertheless, the roles of most WRKY factors and their possible roles in JA signaling are largely unclear, particularly in non-model plants, including pepper.

Pepper serves as one of the important vegetables of global significance due to its multifaceted contributions to agriculture and health. As a member of the Solanaceae family grown in tropical and subtropical regions, pepper frequently suffers from soil-borne pathogens, including *Ralstonia solanacearum*. Bacterial wilt, triggered by strains of the *Ralstonia solanacearum* species complex (RSSC), results in significant economic damage to various crops globally. [18]. Utilizing disease-resistant varieties is the most fundamental strategy to control the disease in pepper production. Understanding the disease-resistance mechanisms of peppers can provide a theoretical basis for the genetic improvement of disease-resistant varieties. JA signaling was suggested to function in the regulation of plant defense against *R. solanacearum*. For example, the cell wall protein fraction (CWP)-induced defense system against *R. solanacearum* was regulated by JA-mediated signaling pathways [19]. The resistance gene *AhRRS5* from peanut, with an up-regulated expression pattern upon methyl jasmonate (MeJA) treatment, positively regulates the resistance of peanut against *R. solanacearum* infection via activating the transcriptional expression of marker genes associated with JA, SA, and ET signals [20]. Various WRKY members in host plants were also found to be induced by the application of exogenous MeJA and take positive roles in the defense response of the host in response to *R. solanacearum* infection, including CaWRKY6, CaWRKY22, and CaWRKY58 in pepper, and NtWRKY50 in tobacco [21,22,23,24]. However, the role of other members of pepper WRKY in the JA signaling-mediated resistance of pepper plants against *R. solanacearum* needs to be further explored, especially the mechanism by which WRKY members regulate the expression of JA-responsive genes. Here, we report that *CaWRKY22b* is especially up-regulated by the exogenous application of MeJA and confers resistance to *R. solanacearum* infection in pepper via directly regulating the expression of JA-responsive *CaDEF1*.

## 2. Results

### 2.1. Sequence Analysis of CaWRKY22b

**Figure 1:**
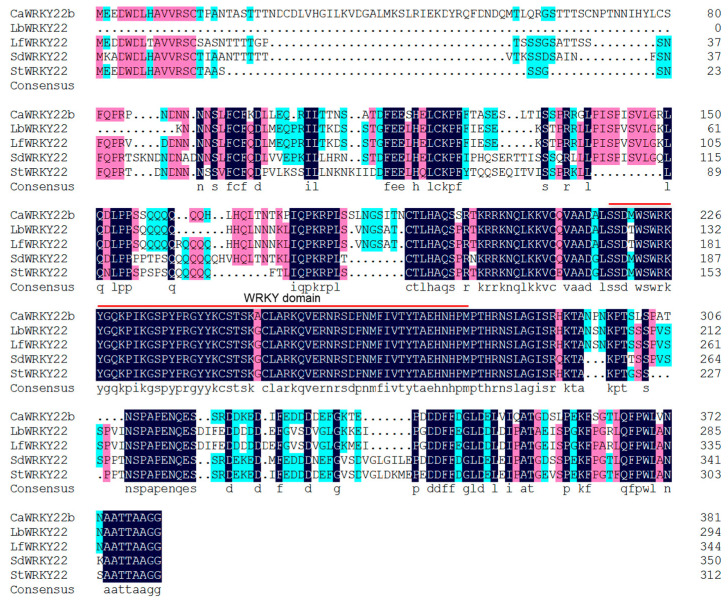
Amino acid sequence arrangements of CaWRKY22b protein and its orthologs from other plant species. Amino acid sequence alignments of CaWRKY22b and its orthologs from *Lycium barbarum* (LbWRKY22), *Lycium ferocissimum* (LfWRKY22), *Solanum dulcamara* (SdWRKY22), and *Solanum tuberosum* (StWRKY22). The conserved WRKY domain is indicated by red line.

In an earlier study of the transcriptome of pepper in response to *Ralstonia solanacearum* based on RNA-seqs, a gene encoding WRKY transcription factor (TF) and exhibiting an up-regulation pattern aroused our attention. We found the WRKY (LOC107845803) shared the highest amino acid sequence identity with WRKY22 among all WRKYs in *Arabidopsis thaliana* (*Arabidopsis*). We designated it as CaWRKY22b to distinguish it from CaWRKY22, a positive regulator of pepper immunity against *R. solanacearum* [21]. Nucleotide sequence analysis revealed that CaWRKY22b was 1559 bp in length, comprising 137 bp of 5′ untranslated region (UTR), 1146 bp of open reading frame (ORF), and 273 bp of 3′ untranslated region. The ORF of CaWRKY22b was predicted to encode a protein of 381 amino acid residues in length, harboring a typical WRKY domain with 60 amino acids (Figure 1). The relative molecular mass and theoretical isoelectric point (pI) of the predicted protein were 42.92 kDa and 6.42, respectively. CaWRKY22b shares 81.98, 81.61, 82.18, and 87.09% amino acid identity with WRKY22 from *Lycium barbarum* (LOC132632597), *Lycium ferocissimum* (LOC132053534), *Solanum dulcamara* (LOC129876496), and *Solanum tuberosum* (LOC107062867), respectively (Figure 1).

### 2.2. Transcript Levels of CaWRKY22b Were Induced by R. solanacearum Infection and Exogenous MeJA Application

**Figure 2:**
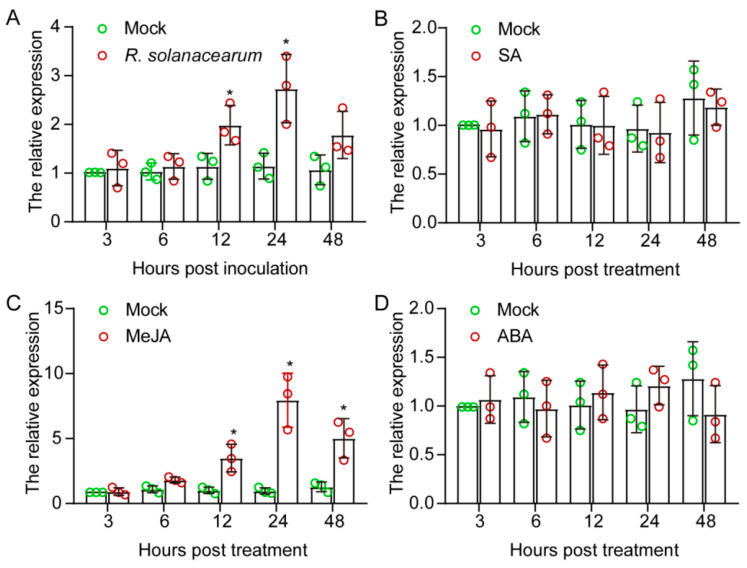
Expression of *CaWRKY22b* was up-regulated against pathogen infection and exogenous MeJA application. (**A**) Up-regulation of *CaWRKY22b* transcript in stems of pepper plants inoculated with *Ralstonia solanacearum* at the root for 48 h. (**B**–**D**) The levels of *CaWRKY22b* transcript in pepper leaves sprayed with 1 mM SA (**B**), 100 μM MeJA (**C**), and 100 μM ABA (**D**). (**A**–**D**) Hollow dots with different colors represent data from three independent experiments. *CaWRKY22b* transcript levels in *R. solanacearum* inoculated- or hormone-treated pepper plants were compared with those in mock-treated control plants, which were set to a relative expression level of “1” at 3 h post-treatment. All treatments were repeated thrice with similar results. The pepper *CaACTIN* and *18S ribosomal RNA* were used as internal controls. Asterisks indicated significant differences as determined by a two-tailed *t*-test (* *p* < 0.05).

To confirm the results of the transcriptome that *CaWRKY22b* transcripts were significantly induced in response to *R. solanacearum* infection, a quantitative RT-PCR was performed using stems of pepper plants challenged with *R. solanacearum* by root irrigation. The results showed that *CaWRKYK22* transcript in pepper stem began to be up-regulated at 12 h post-inoculation (hpi) with a 1.73-fold increase, and the increment reached a peak at 24 hpi with a 2.39-fold increase (Figure 2A). The study by Du et al. revealed that *CaWRKY22b* in hypocotyl was also transcriptionally induced by 1.3-fold at 24 hpi [25]. Phytohormones, including SA, JA, and ABA, serve as vital signaling molecules and play an important role in regulating the expression of defense-associated marker genes during plant-pathogen interaction. To validate the potential role of CaWRKY22b in signaling pathways mediated by these phytohormones, the transcript abundances of *CaWRKY22b* in functional leaves of four-leaf pepper plants sprayed with exogenous hormones were determined by quantitative RT-PCR (Figure 2B–D). The results demonstrated that the *CaWRKY22b* transcript was induced by MeJA treatment, as *CaWRKY22b* mRNA levels in the leaves of MeJA-treated plants were significantly higher than those in mock-treated plants at 6, 12, and 24 h post-treatment (hpt) (Figure 2C). Of note, no significant inductions of *CaWRKY22b* transcript were detected in pepper plants exposed to SA and ABA treatments (Figure 2B,D). Taken together, these results show that CaWRKY22b might participate in pepper defense response against *R. solanacearum* and the JA signaling pathway.

### 2.3. Subcellular Localization of CaWRKY22b in Nicotiana benthamiana

**Figure 3:**
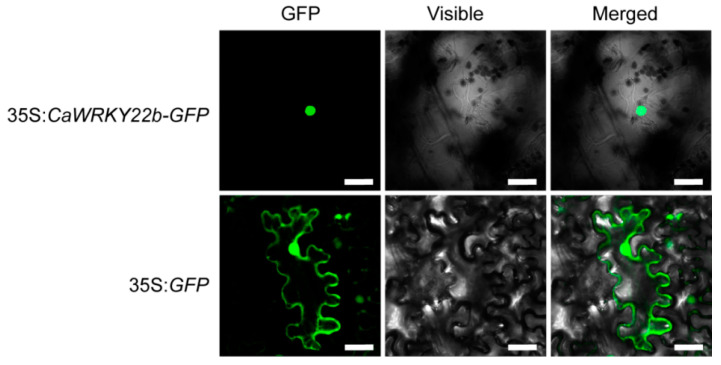
Subcellular localization of CaWRKY22b in *Nicotiana benthamiana. Agrobacterium tumefaciens* GV3101 cells carrying *35S:CaWRKY22b-GFP* and empty vector (35S:GFP) were infiltrated into *N. benthamiana*, respectively. At 48 h post-infiltration, the leaves transiently expressing *CaWRKY22b-GFP* and *GFP* were harvested for fluorescence detection using a laser confocal microscope. Bar = 50 μm.

To further dissect the subcellular localization of CaWRKY22b, *Agrobacterium*-mediated transient expression of *CaWRKY22b* fused to the green fluorescent protein (GFP) was performed in leaves of *N. benthamiana*, and *35S:GFP* construct was served as a control. At 48 h post agroinfiltration (hpa), the infiltrated leaves were harvested for the detection of GFP signals using a laser scanning confocal microscope. The results revealed that when fused to the N-terminus of the GFP protein driven by the *CaMV35S* promoter, CaWRKY22b-GFP exhibited a robust GFP signal within the nuclei (Figure 3). For the control, GFP signals throughout the cells were observed, including the plasma membrane, cytoplasm, and nuclei (Figure 3). To sum up, like most WRKY members, the product of CaWRKY22b is mainly distributed in the nucleus, exhibiting the general characteristics of transcription factors.

### 2.4. Transient Transformation of CaWRKY22b Triggered an Intensive Hypersensitive Response-like Cell Death

**Figure 4:**
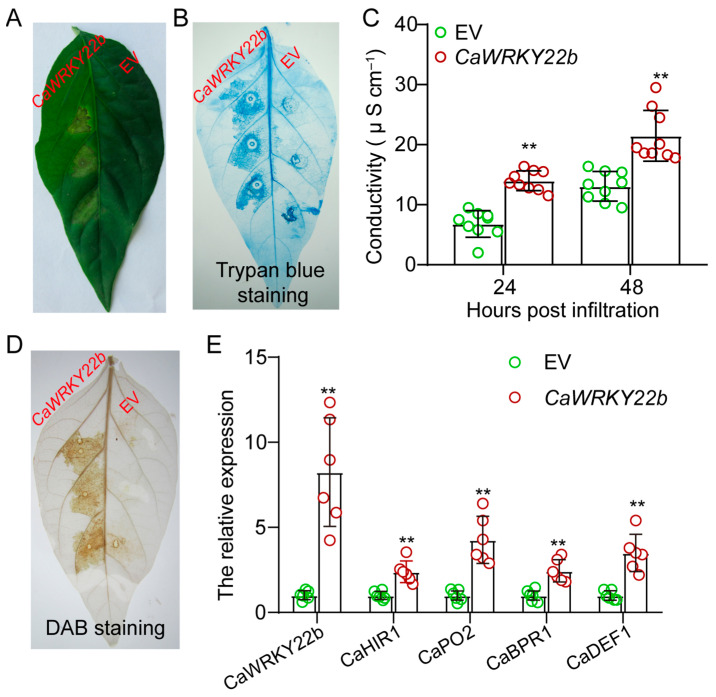
*Agrobacterium*-mediated transient expression of *CaWRKY22b* in leaves induced intensive hypersensitive response-like cell death. (**A**) The phenotype of pepper leaves transformed with *CaWRKY22b* and empty vector (EV). *Agrobacterium tumefaciens* cells carrying *35S:CaWRKY22b* and *35S:00* (EV) were infiltrated into the leaves of pepper plants. At 72 h post infiltration (hpi), the HR-like cell death was detected and captured with a camera. (**B**) The trypan blue staining was performed to determine the cell death in pepper leaves triggered by the transient expression of *CaWRKY22b* at 48 hpi. (**C**) The electrolyte leakages were measured at 24 and 48 h post infiltration in pepper leaves transiently expressing *CaWRKY22b* and EV. (**D**) At 48 hpi, the DAB staining was carried out to evaluate the H_2_O_2_ accumulation in pepper leaves transiently transformed with *CaWRKY22b* and EV. (**E**) The transcript abundances of defense- and MeJA-responsive marker genes, including *CaHIR1*, *CaPO2*, *CaBPR1*, and *CaDEF1*. Hollow dots with different colors represent six biological replicates from two independent experiments. The expressions of the tested genes in pepper leaves transiently expressing EV were set to a relative expression of “1”. The pepper *CaACTIN* and *18S ribosomal RNA* were used as internal controls. Asterisks indicated significant differences as determined by a two-tailed *t*-test (** *p* < 0.01).

It is well established that hypersensitive response (HR)-like cell death plays a vital role in plant immunity, including limiting the propagation of pathogens and activating the defense response and the induction of systematic-acquired resistance. In order to ascertain if CaWRKY22b plays a role in inducing HR-like cell death, *CaWRKY22b* driven by *CaMV35S* promoter was transiently expressed in the leaves of pepper plants, followed by the observation of cell death. Forty-eight hours after agroinfiltration, a pronounced cell death was noticed in the pepper leaves that were transformed with *CaWRKY22b* (Figure 4A). However, no visible cell death was detected in leaves expressed with the empty vector (EV). Cell death was further assessed using trypan blue staining and by measuring the electrolyte leakage. Increased numbers of dark blue zones and higher conductivity were observed in *CaWRKY22b*-expressed leaves compared with those expressing empty vector, indicating that the CaWRKY22b product is capable of triggering cell death in pepper leaves (Figure 4B,C). Additionally, transient expression of *CaWRKY22b* induced H_2_O_2_ accumulation in the infiltrated site, as confirmed by the dark brown color of diaminobenzidine (DAB) staining in *CaWRKY22b*-transformed leaves compared with the EV leaves (Figure 4D). To determine whether *CaWRKY22b* expression induces a defense response in pepper leaves, the transcript abundances of defense- and hormones-associated marker genes were detected, including *CaHIR1* (hypersensitive-induced reaction protein gene) [26], *CaPO2* (extracellular peroxidase) [27], *CaBPR1* (*basic PR-1*) [28], and *CaDEF1* (JA-responsive gene) [29]. To exclude the possibility that transient overexpression of *CaWRKY22b* inhibits the expression level of endogenous *CaWRKY22b*, the total expression of *CaWRKY22b*, including transgenic and endogenous *CaWRKY22b,* was detected. The result of quantitative RT-PCR indicated that the expression level of total *CaWRKY22b* detected in pepper leaves transiently expressing *CaWRKY22b* was significantly higher than that of empty vector (Figure 4E), suggesting *CaWRKY22b* was successfully transiently expressed in pepper leaves. Additionally, the transcript accumulations of all the tested marker genes were significantly induced by the transient expression of *CaWRKY22b* (Figure 4E). The results above indicated that CaWRKY22b plays a positive role in plant cell death, probably in a manner associated with JA signaling.

### 2.5. Reducing the Expression of CaWRKY22b Comprised the Pepper’s Resistance to R. solanacearum

**Figure 5:**
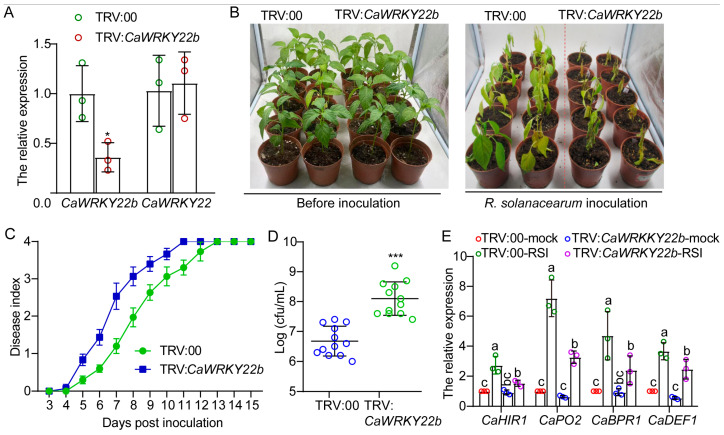
*CaWRKY22b* silencing in pepper plants attenuates its immunity against *R. solanacearum* inoculation. (**A**) The relative expression of *CaWRKY22b* and *CaWRKY22* in leaves of *CaWRKY22b*-silenced (TRV:*CaWRKY22b*) and unsilenced pepper plants (TRV:00). (**B**) Disease symptoms in pepper plants at eight days post inoculation with *R. solanacearum* by root irrigation. (**C**) Progression of bacterial wilt ranging from 3 to 15 days in *CaWRKY22b*-silenced and unsilenced pepper plants challenged with *R. solanacearum* by root irrigation. Data represent three replicate experiments, and 16 plants were calculated for each experiment. Bars represent standard error. (**D**) Bacterial growth in the stems of pepper plants inoculated with *R. solanacearum* by stem injection. A total of 5 μL of bacterial suspension (10^6^ CFU/mL) was injected into the stem of pepper plants, and the xylem sap was harvested from the infected plant for bacterial quantification at 2 days post-inoculation. Hollow dots with different colors represent 12 biological replicates from two independent experiments. Asterisks indicate significant differences (*** *p* < 0.001, two-tailed *t*-test). (**E**) The relative expression of defense- and MeJA-associated marker genes in stems of pepper plants at 48 h post-*R. solanacearum* inoculation by root irrigation. RSI, *R. solanacearum* inoculation; mock, treated with sterilized ddH_2_O. Hollow dots with different colors represent data from three independent experiments. (**A**,**D**,**E**) * *p* < 0.05, *** *p* < 0.001 (two-tailed *t*-test) and different letters indicate significant differences as analyzed using Fisher’s protected LSD test (*p* < 0.05).

The results show that *CaWRKY22b* transcripts in the stem of pepper plants are up-regulated in response to *R. solanacearum* infection and CaWRKY22b positively regulated plant cell death, suggesting that CaWRKY22b might function in pepper immunity against *R. solanacearum*. To validate this, the transcript level of *CaWRKY22b* was knocked-down in pepper plants using a virus-induced gene silencing (VIGS) approach, and the plants silenced with *CaWRKY22b* were then subjected to *R. solanacearum* infection. The silencing efficiency of *CaWRKY22b* in silenced pepper plants was determined using a quantitative RT-PCR (Figure 5A). The transcript level of *CaWRKY22b* was reduced in the stems of TRV:*CaWRKY22b* pepper plants (Figure 5A), indicating that *CaWRKY22b* was effectively silenced in pepper. No significant difference in *CaWRKY22b* transcript was detected between TRV:*CaWRKY22b* and TRV:00 pepper plants, suggesting the specific silencing of *CaWRKY22b* in our VIGS assay (Figure 5A). Of note, we did not observe any difference in morphology and growth between *CaWRKY22b*-silenced and unsilenced pepper plants (Figure 5B, left). FJC100301, a highly virulent strain of *R. solanacearum,* was employed to inoculate VIGS-treated pepper plants via root irrigation to ascertain the impact of *CaWRKY22b* silencing on the pepper’s resistance against *R. solanacearum*. Upon being challenged with *R. solanacearum*, the *CaWRKY22b*-silenced pepper displayed more serious bacterial wilt symptoms than the pepper silenced with an empty vector (Figure 5B, right). By contrast, higher disease indices were detected in *CaWRKY22b*-silenced pepper (Figure 5C). We also detected whether the silencing of *CaWRKY22b* influences the growth of *R. solanacearum* in pepper. The result of the CFU assay indicated that *CaWRKY22b* silencing prompts the propagation of *R. solanacearum* in pepper stems (Figure 5D).

To further unravel the possible mechanism of CaWRKY22b-mediated resistance of pepper to *R. solanacearum*, the effects of *CaWRKY22b* silencing on the transcript levels of defense- (*CaHIR1*, *CaPO2*, and *CaBPR1*) and JA-related marker genes (*CaDEF1*) were also investigated. The results of quantitative RT-PCR showed that the expression of the tested genes in the stem of unsilenced pepper plants was up-regulated in response to *R. solanacearum*. However, the increments were all significantly abolished by *CaWRKY22b* silencing (Figure 5E). Taken together, the aforementioned results imply that CaWRKY22b positively regulates pepper’s resistance to *R. solanacearum*.

### 2.6. CaWRKY22b Modulates the Expression of JA-Responsive CaDEF1 by Directly Binding to Its Promoter

**Figure 6:**
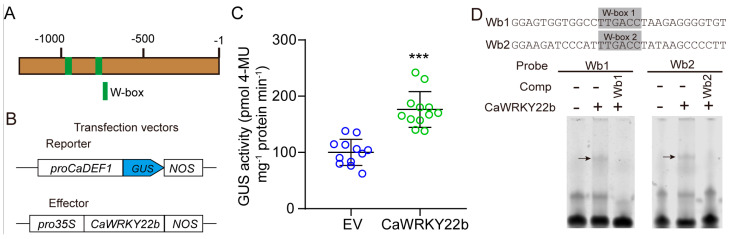
(**A**) The location of typical W-boxes in the promoter of *CaDEF1*. (**B**) Schematic diagram of the effector and reporter constructs used in detecting the activity of *CaDEF1* promoter. (**C**) GUS activities in pepper leaves co-transformed with the tested effector and reporter constructs. *Agrobacterium tumefaciens* cells carrying *pCaDEF1:GUS* and *35S:CaWRKY22b* were co-infiltrated into the leaves of 6-week-old pepper plants. At 48 h post infiltration, the infiltrated leaves were harvested for the quantification of GUS activities. Hollow dots with different colors represent 12 biological replicates from three independent experiments. *** indicated extreme significant difference, as determined by a two-tailed *t*-test (*p* < 0.001). (**D**) EMSAs showed that the CaWRKY22b protein both bound to the two typical W-boxes contained in *CaDEF1* promoter. As competitors, 100-fold excess of unlabeled probes were used. Wb1/Wb2, W-box 1/W-box 2 in the *CaDEF1* promoter; Comp, cold competitors; black arrows indicate specific shifts.

Finding that transient overexpression of *CaWRKY22b* induced the expression of JA-responsive *CaDEF1* leads us to hypothesize that CaWRKY22b might play a crucial role in the up-regulation of JA-responsive *CaDEF1* during defense response against *R. solanacearum*. Since the expression of a target gene is strictly modulated by its native promoter, the promoter regions of *CaDEF1* were examined using the PlantCARE database (http://bioinformatics.psb.ugent.be/webtools/plantcare/html/, accessed on 12 May 2023), which provides information on *cis*-acting regulatory elements. The analysis results indicated that two W-boxes, potential binding sites of WRKY TFs, were contained in the *CaDEF1* promoter (Figure 6A). We hypothesized that CaWRKY22b could regulate the promoter activity of *CaDEF1* to modulate its expression level. To verify this hypothesis, 1327 bp-long sequences of the *CaDEF1* promoter were cloned to generate the β-glucuronidase (GUS)-base reporter construct (*pCaDEF1:GUS*) (Figure 6B). *CaWRKY22b* fused to *GFP-tag* was placed under the control of the *CaMV35S* promoter to construct an effector construct (*35S:CaWRKY22b-GFP*) (Figure 6B). The two constructs generated were simultaneously co-transformed into the leaves of pepper plants and the GUS activities were measured to evaluate the effect of *CaWRKY22b* expression on the promoter activity of *CaDEF1*. The results of GUS activity quantification indicated that overexpression of *CaWRKY22b* significantly enhanced the activities of the *CaDEF1* promoter since the GUS activity in leaves transiently co-expressing *pCaDEF1:GUS* and *35S:CaWRKY22b-GFP* is higher than in leaves co-expressing *pCaDEF1:GUS* and an empty vector (Figure 6C). We further ask how CaWRKY22b modulates the promoter activity of *CaDEF1*. The fact that two W-boxes were contained in the *CaDEF1* promoter leads us to hypothesize that CaWRKY22b might regulate the *CaDEF1* promoter by directly binding to the W-box(es). To validate this, electrophoretic mobility shift assays (EMSAs) were conducted utilizing cy5-labeled probes of the *CaDEF1* promoter fragments that contain W-box (30 bp) and the purified CaWRKY22b protein, which was expressed from *Escherichia coli*. The cy5-labeled W-box 1 and W-box 2 probes were incubated with CaWRKY22b protein in vitro. Both the probes led to significant shifts, and the shifts efficiently competed with a 100-fold excess of unlabeled W-box 1 or W-box 2, suggesting a direct interaction between CaWRKY22b and the tested W-boxes (Figure 6D). Collectively, the results revealed that CaWRKY22b might modulate the expression of JA-responsive *CaDEF1* by directly binding to the W-boxes of its native promoter.

## 3. Discussion

### 3.1. CaWRKY22b Functions as a Positive Regulator in Defense Response of Pepper Plant against R. solanacearum

It is widely recognized that WRKY family members play a crucial role in plants’ defense response to adverse environmental conditions, such as biotic stress. The expression of numerous *WRKY* genes is generally induced by pathogen attacks. In *Arabidopsis*, 49 of 72 WRKY members were found be to differentially regulated in response to the infection of an avirulent bacterial pathogen or the application of exogenous SA [30]. In our present study, we found *CaWRKY22b* transcripts to be significantly induced by the infection of *R. solanacearum* and the application of exogenous MeJA (Figure 2) and knock-down of *CaWRKY22b* in pepper plants significant attenuate its resistance to *R. solanacearum* (Figure 5). It is reported that various WRKY members in pepper plants were transcriptionally induced by the challenge of *R. solanacearum* and play vital roles during the defense response, including CaWRKY6, CaWRKY22, CaWRKY27, CaWRKY40, etc., [21,23,24,31,32].

Transcript accumulations of HR-, H_2_O_2_ burst-, and basal defense-related genes are typically observed during defense response [31]. In pepper, *CaHIR1*, *CaPO2*, and *CaBPR1* are commonly up-regulated against *R. solanacearum* [21,33,34]. In the present study, transient overexpression of *CaWRKY22b* (Figure 4) in pepper leaves up-regulated transcript levels of *CaHIR1*, *CaPO2*, *CaBPR1*, and *CaDEF1*. Consequently, the immunity of pepper plants against *R. solanacearum* mediated by CaWRKY22b is likely based on the transcription of serial HR- and defense-related genes promoted by CaWRKY22b. Similarly, the immunity conferred by CaWRKK40 against *R. solanacearum* is probably based on the transcription factor’s enhancing effect on the transcription of a diverse set of HR- and defense-related genes [31].

The reduction in *CaWRKY22b* expression attenuated the immunity of pepper plants against *R. solanacearum* infection, as evidenced by an increased disease index and accelerated growth of *R. solanacearum* in the *CaWRKY22b*-silenced pepper plants (Figure 5). This implies that CaWRKY22b might play a positive role in pepper immunity against *R. solanacearum*. Additionally, transient expression of *CaWRKY22b* triggered intensive H_2_O_2_ accumulation and hypersensitive response-like cell death (Figure 4). We hypothesize that during the defense response of pepper against *R. solanacearum* interaction, *R. solanacearum* induced the transcript accumulation of *CaWRKY22b*. In turn, the up-regulation of *CaWRKKY22b* triggers an H_2_O_2_ burst and thus the hypersensitive cell death to limit the propagation of *R. solanacearum*. However, the mechanism by which CaWRKY22b participates in the regulation of the ROS burst and HR-like cell death needs to be further elucidated.

### 3.2. CaWRKY22b Functions in JA Signalling by Regulating the Expression of JA-Responsive CaDEF1

The production of numerous hormones, including SA, ABA, and JA, is generally induced upon the attack of the pathogen. The hormones are proven to regulate plant immunity by activating the expression of defense-associated genes, including WRKY members. In our present study, we found that the *CaWRKY22b* transcript is significantly induced by the application of exogenous MeJA (Figure 2). However, no transcriptional alteration of *CaWRKY22b* was detected in response to the treatments of exogenous SA and ABA (Figure 2), suggesting CaWRKY22b may specifically participate in the JA signaling pathway. It is well established that JA signaling is utilized by the host plants to promote defense response to fight against necrotrophic pathogens, including *R. solanacearum*. Furthermore, *CaWRKY22b* transient expression in pepper leaves triggered the transcript accumulation of JA-responsive *CaDEF1* (Figure 4E). We hypothesize that CaWRKY22b may participate in JA signaling by modulating the expression of *CaDEF1*. Indeed, transient overexpression of *CaWRKY22b* significantly induced the activity of *CaDEF1* promoter and CaWRKY22b is capable of binding to the promoter of *CaDEF1* directly (Figure 6). Interestingly, the transcript level of *CaDEF1* was reported to be regulated by many other WRKY members, including *CaWRKY22*, *CaWRKY30*, and *CaWRKY3* [21,35,36]. However, how these different WRKY members regulate the expression of *CaDEF1* in a coordinated manner remains to be further studied.

Collectively, our result suggests that CaWRKY22b positively regulated pepper immunity against *R. solanacearum* in a manner associated with JA signaling, probably by modulating the expression of JA-responsive *CaDEF1*.

## 4. Materials and Methods

### 4.1. Plant Materials, Hormones Application, and Pathogen Inoculation

Seeds of the HN42 pepper inbred line, obtained from Fujian Agriculture and Forestry University, were sown in a commercial soil mix consisting of peat moss and perlite in a 2:1 ratio by volume. The seeds were planted in plastic pots and maintained in a greenhouse at 25 °C, 60–70 mmol photons m^−2^ s^−1^, and a relative humidity of 70%, under a 16/8 h photoperiod.

For the application of exogenous hormones, leaves of pepper plants at the four-leaf stage were sprayed with 1 mM salicylic acid (SA), 100 μΜ methyl jasmonate (MeJA), and 100 μΜ abscisic acid (ABA). Pepper plants treated with sterilized ddH_2_O were used as a control.

The inoculation of *R. solanacearum* through soil-drenching was carried out as previously described [37]. Essentially, cultured cells of *R. solanacearum* (FJC100301) were collected via centrifugation, resuspended in sterilized distilled water, and adjusted to a concentration of 10^8^ CFU/mL. Before inoculation, the roots of fully leaf-expanded pepper plants were slightly damaged by inserting a knife into the soil near the root area three times. After inoculation, the plants were placed in a chamber set at 28 °C and 75% humidity. For bacterial quantification, a 2.5 μL sample of xylem sap was harvested from each plant at 48 h post-inoculation for CFU counting. The disease index was assessed daily on a scale from 0 to 4, in accordance with the previous description [38].

### 4.2. Quantitative Real-Time PCR

The extraction of total RNA from pepper plants was carried out using the TRIZOL reagent (Aidlab, Beijing, China) and the genomic DNAs were digested using DNAse (Aidlab, Beijing, China), according to a previous study [38]. The isolated total RNAs were subsequently converted into complementary DNAs (cDNAs) through reverse transcription assays using MMLV reverse transcriptase (Thermo Scientific, Rockford, IL, USA). The obtained cDNA was then diluted tenfold and used in quantitative RT-PCR assays, which were performed with a commercial SYBR premix (Vazym, Nanjing, China), adhering to the guidelines provided in the kits. The mRNA levels of *CaACTIN* (GQ339766) and *18S ribosomal RNA* (EF564281) served as references to normalize the relative expression levels of the tested genes [39]. To ensure reliability, each biological sample underwent three technical replicates for analysis.

### 4.3. Subcellular Localization

The assay to determine subcellular localization of CaWRKY22b was conducted as outlined in the previous study [40]. In summary, *Agrobacterium tumefaciens* cells of the GV3101 strain, which carries *35:CaWRKY22b-GFP*, were transiently expressed in the leaves of *N. benthamiana* plants at 5 weeks old. At 48 h post-transformation, the infiltrated leaves were collected for the detection of fluorescent signals using a laser confocal microscope (SP8, Leica, Wetzlar, Germany). The emission and excitation wavelengths of GFP were set at 510–520 nm and 488 nm, respectively.

### 4.4. Agrobacterium-Mediated Transient Expression of CaWRKY22b

The procedure for the *Agrobacterium*-mediated transient expression assay was performed following a previously established method with minor alterations [41]. The full ORF of *CaWRKY22b* was amplified from pepper cDNA using the primers listed in Table S1. The amplified *CaWRKY22b* fragment flanked with attB cloning sites was cloned into the satellite vector pDONR207 (Thermo Scientific, Waltham, MA, USA) via BP reaction and transferred to the destination vector via LR reaction (Thermo Scientific, Waltham, MA, USA) to generate *35S:CaWRKY22b*. Afterward, *Agrobacterium tumefaciens* cells of the GV3101 strain containing the *35S:CaWRKY22b* construct were expressed in the expanded leaves of pepper plants through agroinfiltration using a needleless syringe. After the infiltration, the plants were kept in a controlled environment. Leaf samples were then collected at 24–48 h post agroinfiltration for further analyses, including histochemical staining, electrical conductivity measurements, and extraction of total RNA.

### 4.5. Virus-Induced Gene Silencing (VIGS) in Pepper Plants

The VIGS system, which is based on the tobacco rattle virus (TRV), was employed to reduce *CaWRKY22b* expression in pepper plants, following a methodology outlined in a previous study [42]. The specific segment for *CaWRKY22b* silencing, whose specificity was verified at https://solgenomics.net/tools/blast/ (accessed on 21 September 2022), underwent amplification with the primers listed in Table S1. This amplified segment was subsequently cloned into the satellite vector pDONR207. After sequencing to ascertain the correctness of the segment, the fragment was integrated into the pTRV2 vector, resulting in TRV:*CaWRKY22b*. *Agrobacterium tumefaciens* GV3101 cells, containing either pTRV1 and pTRV2:00 (empty vector) or pTRV2:*CaWRKY22b*, were adjusted to an OD595 of 0.6 and blended in a 1:1 ratio. This mixture was then infiltrated into cotyledons of pepper plants aged 2–3 weeks. These treated plants were maintained at 25 °C under a cycle of 16 h of light and 8 h of darkness. After undergoing 5–6 weeks of VIGS treatment, the plants were used for further studies.

### 4.6. Histochemical Staining

Histochemical staining procedures, inclusive of DAB and trypan blue staining, were carried out as previously described with minor adjustments [43]. The reagents utilized included 1 mg/mL diaminobenzidine (Sigma-Aldrich, St. Louis, MO, USA) for DAB staining and a mixture of lactophenol-ethanol trypan blue solution (10 mL lactic acid, 10 mL glycerol, 10 g phenol, 30 mL absolute ethanol, and 10 mg trypan blue dissolved in 10 mL distilled deionized water) for trypan blue staining, respectively. Following the DAB staining, the leaf samples were cleared by boiling in [lactic:glycerol:absolute ethanol (1:1:3, V:V:V)] and rinsed overnight in absolute ethanol solution to remove excess dye. In contrast, after trypan blue staining, leaf destaining was performed using a chloral hydrate solution (dissolving 2.5 g of chloral hydrate in 1 mL of distilled water). Images representative of the results were then captured using a light microscope (Leica, Wetzlar, Germany).

### 4.7. The Measurement of GUS Activity

β-Glucuronidase activity was measured using fluorometric assays [44]. Briefly, the leaves transiently expressing *GUS* were homogenized and suspended in extraction buffer [50 mM NaH_2_PO_4_, pH 7.0, 10 mM EDTA, 0.1% Triton X-100, 0.1% (*w*/*v*) sarcosyl (*w*/*v*), and 10 mM β-mercaptoethanol]. The supernatant was harvested by centrifugation for 10 min at 12,000× *g* at 4 °C, and the GUS activity was measured according to the methods described by Jefferson [44].

### 4.8. Electrophoretic Mobility Shift Assay

Electrophoretic mobility shift assays were performed as previously described [37]. Briefly, 30 bp fragments of *CaDEF1* promoter containing W-box 1 or W-box 2 were labeled with cy5 and were synthesized to generate probes. CaWRKY22b-GST protein was expressed and purified from *E. coli* cells. Purified CaWRKY22b protein was incubated with the cy5-labeled probes in a 5× binding buffer [(200 mM Tris–HCl, pH 7.5, 375 mM KCl, 6.25 mM MgCl_2_, 25% glycerol, and 1 mM DTT)]. The mixture was kept on ice for 45 min and separated by running on a polyacrylamide gel, followed by scanning on an Odyssey@ CLX instrument (LI-COR, Lincoln, NE, USA).

### 4.9. Statistical Analyses

The statistical significance between multiple groups was represented by distinct letters (*p* < 0.01), as determined by Fisher’s least significant difference (LSD) test. The statistical significance between pairs of groups was denoted by either a single asterisk (* *p* < 0.05), double asterisks (** *p* < 0.01), or three asterisks (*** *p* < 0.001), determined by the two-tailed *t*-test.

## Data Availability

The original contributions presented in the study are included in the article/Supplementary Materials, further inquiries can be directed to the corresponding author.

## Supplementary Materials

The following supporting information can be downloaded at: https://www.mdpi.com/article/10.3390/plants13152081/s1, Table S1: Oligonucleotides used in this study.
